# Ovarian antral follicle dynamics from birth through reproductive maturity: a mini-review

**DOI:** 10.3389/fendo.2026.1862922

**Published:** 2026-06-17

**Authors:** Hannah Lamar, Rodolfo Cardoso, Heidi Vanden Brink

**Affiliations:** 1 Department of Nutrition, Texas A&M University, College, Station, TX, United States; 2 Department of Animal Science, Texas A&M University, College, Station, TX, United States

**Keywords:** adolescence, animal model, antral follicle, human, ovary, puberty

## Abstract

The transition from quiescence to a mature hypothalamic-pituitary-ovary (HPO) axis is a complex window of female reproductive maturation spanning puberty and the early postmenarcheal years. In reproductive maturity, the ovaries integrate reproductive and metabolic endocrine signals to coordinate both ovulatory and anovulatory waves of antral follicle development. Reproductive development towards a mature reproductive axis during adolescence is essential for optimal physiology and long-term reproductive health. However, abnormal reproductive maturation during this window is increasingly prevalent, highlighting the need to better understand the physiological and pathophysiological phases of reproductive maturity leading to ovarian antral follicle wave dynamics that define optimal reproductive maturity. In this mini-review, we will summarize literature in both humans and ruminant animal models to describe what is known – and unknown — of the endocrinology and morphology of follicle wave dynamics and follicular growth profiles from birth through reproductive maturity.

## Introduction

Hypothalamic-pituitary-ovary (HPO) axis maturity in women is characterized by the coordination of two or three waves of antral follicle development across ovarian cycle (1). How the HPO axis transitions from the immaturity of childhood through puberty to the mature female remains undefined. Appreciating the comparative reproductive physiology of female ruminants and humans (2–5), this mini-review summarizes literature in select large animal models and humans of the endocrinology and morphology of follicle wave emergence during reproductive axis maturation. The review begins with an overview of the ruminant animal models, followed by insights gained from existing human evidence with a proposed hypothesis of anticipated wave emergence dynamics.

## Insights from the ruminant animal model

**Figure 1:**
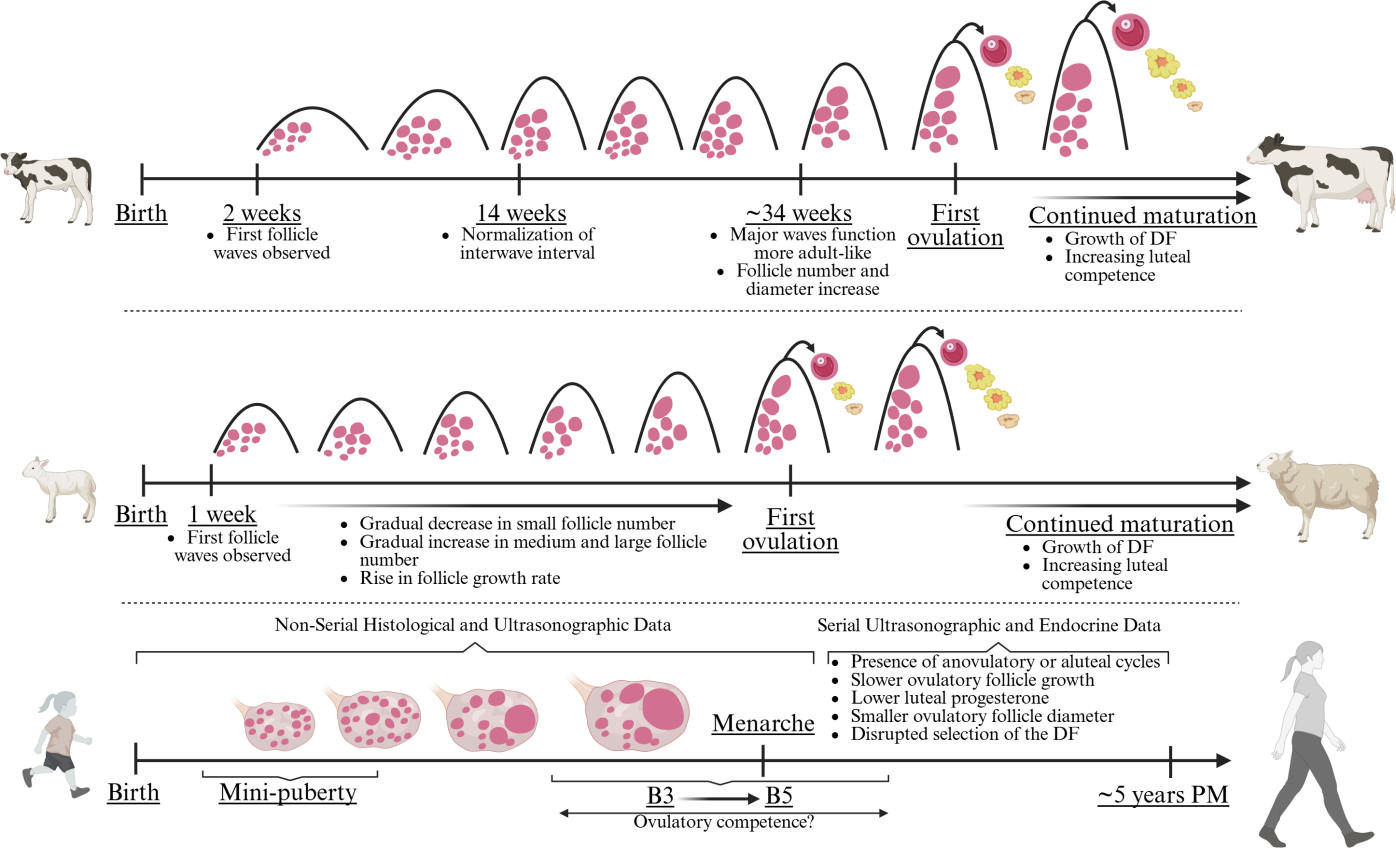
Graphical summary of the trajectory of ovarian follicle wave dynamics and ovarian morphology, where data exist, from birth through reproductive maturity in bovine (6, 7, 8, 10), ovine (4, 17), and human (21, 23, 31) models. Created in BioRender. Wester Lamar, H. (2026) https://BioRender.com/rf5r4co.

Follicle dynamics spanning postnatal to adulthood are widely documented in ruminants and share fundamental wave-like endocrine and follicle dynamics as have been observed in adult women (2, 5). The larger anatomical presentation of the ovaries and follicles allows for clear assessment of follicle dynamics versus rodents and thus ruminants have been postulated to serve as animal models for human physiology as previously discussed (2). However, an important limitation persists in the translational potential between ruminants and humans that should be considered. First ovulation marks the start of puberty in animal models, whereas menarche is often used as a proxy for this major physiological event in adolescents and thus differentiates the prepubertal and postpubertal mammal. This distinction is critical as we consider the timeline towards reproductive maturity in animal models versus humans (**Figure 1**).

### Infancy to early calfhood

Follicle and endocrine dynamics of the Hereford breed of cattle from birth through puberty (first ovulation and/or behavioral estrus) at approximately 52 weeks of age (6), have been systematically documented. At 2 weeks of age, Hereford calves already exhibit distinct major wave-like patterns (e.g., formation of a dominant follicle (DF, >8.5–10 mm (7), depending on breed), although with longer inter-wave intervals and smaller maximal DF diameter versus mature females (6). Stabilization of inter-wave intervals of 7 days/wave occurs at 14 weeks of age (6), which is consistent with mature females (6). Across this developmental window of 2–34 weeks of age, maximal DF diameter and number of follicles per wave continuously increase, which is termed the “major phase of postnatal follicular development” (6).

Endocrine patterns present during infancy and early calfhood coincide with evolving follicle dynamics. Circulating estradiol concentrations increase alongside DF growth, reflecting progressive DF maturity as first ovulation nears (6). Within the first 36 weeks of age, follicle stimulating hormone (FSH) and luteinizing hormone (LH) reach maximal concentrations at approximately 6–13 weeks old (6), corresponding with the aforementioned “major phase of postnatal follicular development” (6). The peak in FSH and LH concentration is followed by a progressive decrease in gonadotropin concentrations throughout the remaining weeks leading to puberty. That said, transient increases and decreases in FSH – hallmark for wave emergence (1) – are observed in prepubertal calves as early as 17 days old (6), suggesting prepubertal dependence on hypothalamic-pituitary signaling even in the presence of a still-maturing HPO-axis (6). The progressive decline in overall FSH and LH following the 34-week maximum has been posited to result from DF-mediated suppression of FSH via estradiol and inhibin secretion (6). Maximal follicle diameter continues to increase even in the presence of lower FSH and LH concentrations (6), suggesting the potential for increased follicle sensitivity to gonadotropins following the early peak as the DF continues maturing.

### Calfhood until first ovulation

In 34-52-week-old Hereford females, adult-like major wave-like follicular and endocrine patterns are detected, albeit with some differences from their adult counterparts. Progressive increases in maximal follicle diameter and decreases in overall follicle number occur during the 12 weeks approaching first ovulation (8) which is attributed to increasingly adult-like gonadotropin profiles; daily FSH and LH concentrations were reported to resemble those of postpubertal females, although LH rises were significantly lower versus ovulatory concentrations (8). Despite the smaller LH rise, follicle growth and regression rates are similar to that of adult wave dynamics, and the number of waves per interovulatory interval align with adult dynamics by first ovulation, demonstrating signs of HPO maturation during this prepubertal window (8). Although adult-like patterns emerge, peripubertal maximal DF diameter is still smaller versus postpubertal profiles (e.g., approximately 13.9 mm (8) versus 15–21 mm in late pre- to early puberty versus adult females (9), respectively), indicating that there is some degree of reproductive axis maturation that occurs after first ovulation.

### First and early ovulations in Hereford females

As first ovulation approaches, the LH and estradiol positive feedback mechanism appears intact, resulting in “mini” preovulatory LH surges (e.g., 0.6 ng/mL 72 days before first ovulation versus 3 ng/dL immediately preceding first ovulation) while FSH dynamics remain unchanged (8). Estradiol concentrations preceding and comprising the first ovulatory cycle are comparatively lower than that of the subsequent ovulations, concurrent with the continued rise in maximal DF diameter and competence in subsequent cycles (6, 8). Luteal size following the first ovulation is also smaller and regresses faster versus the second ovulation, resulting in lower serum progesterone secretion that progressively increases during subsequent cycles (8). The shortened corpus luteum (CL) lifespan may be attributed to unique differences in the preovulatory follicle growth profile; early ovulations followed a plateau of the DF at maximal diameter prior to ovulation, which is not observed in adult bovine ovulatory follicle dynamics (10). Whether the plateau reflects a requirement for sustained or cumulative estradiol threshold required to initiate an LH surge is unclear and could reflect either neuroendocrine or ovarian immaturity. Overall, the early first ovulations can be characterized as immature, demonstrated by increased likelihood of subfertility in the earliest versus subsequent ovulatory events (7, 8).

### Characteristics of the ovine model

Like cows, follicle dynamics are observed soon after birth (4) in female lambs and time of first estrus depends on factors including breed, nutritional status, and bodyweight (3, 11), ranging from 6–12 months of age (11–14). Rises in FSH and LH concentrations begin one week after birth along with modest increases in follicle number and size (4), implying early HPO coordination, similar to a mini puberty described in humans (15). Ovarian histological evaluation of Ouled Djellel female sheep collected every 2 weeks between 0–26 weeks old suggests wave-like fluctuations in follicle number and diameter after birth and continuing to puberty (4). Follicle diameters of the growing cohort initially decreased from birth to 1 week old, followed by a rapid increase beginning at 10 weeks old, coupled with prepubertal peaks of LH and FSH concentrations, suggesting ovarian responsiveness to gonadotropins at this prepubertal stage (4). The progressive increase in antral follicular pool diameter continued to week 14, followed by fluctuations in follicle diameter in the weeks thereafter with maximal diameter achieved at week 24 (approximately 7.2 mm) which corresponded with the time at which CL formation, and thus puberty, was observed (4). Accordingly, LH levels rose 6.6-fold at week 24, coinciding with the observed ovulation (4). Ultimately, while wave dynamics cannot be inferred from histological studies, subsequent FSH and LH fluctuations coincided with increases in follicle number and size with the final LH surge resulting in CL formation at 24 weeks old (4), describing wave-like follicle growth and follicle dominance, potentially via estradiol and inhibin A release (16), within the weeks leading up to puberty. Transrectal ultrasonography in female sheep from 145 days old until first ovulation showed significant reductions in small follicle number (3), similar to that observed histologically (4), while the number of medium and large follicles increased with age (3). Indeed, antral follicles ≥ 3mm increased in diameter in a cyclic manner prepubertally in Suffolk x Western White Face ewe lambs (13). Postpubertal sheep may experience slower follicular growth rate compared to adult female sheep (17), although FSH release patterns and peak concentrations appear consistent between groups (18).

## Follicle dynamics in childhood and adolescence

Aligned serial follicle and endocrine studies during this transitory window in humans are lacking. However, cross-sectional histology and endocrinology from birth through puberty (19, 20), along with studies capturing serial endocrinology (21–30), serial ultrasonography (21, 23, 31), or dynamic testing of HPO axis maturity (25, 32–34) offer insights into the progression of ovarian maturation.

### Birth through childhood

Histological and cross-sectional reports from ovariectomized females aged 3–17 years old describe an increase in serum gonadotropins coupled with high total preantral follicle count (19) immediately following birth, which has been termed “mini puberty” (15). After approximately two months of age, there is a progressive decline in circulating gonadotropins and antral follicle number due to pronounced gonadotropin-releasing hormone (GnRH) inhibition (19, 35). Similarly, inhibin A, produced by DFs, is undetectable through approximately 10 years of age (36); however, low fluctuations in FSH and inhibin B are observed during childhood (36). Both homogenous ovaries with no visible follicles (37, 38) and ovaries with antral follicles (20) have been reported, which, taken alongside marginally low inhibin (36), suggests intermittent follicle growth during childhood that could either reflect minor waves or intermittent, uncoordinated follicle development.

### Puberty and premenarche

Cross-sectional studies characterize an increase in ovarian size (39, 40), antral follicle count (41), and anti-Müllerian hormone (AMH) concentrations across the peripubertal years (42, 43). This dynamic trajectory presumably reflects the initiation of gonadotropin-dependent follicular growth and progression towards ovulatory capacity. Approaching puberty, rhythmic secretion of estradiol, inhibin B, FSH, and LH emerges, suggestive of partial ovarian-hypothalamic coordination prior to menarche (25, 36, 44, 45). Drawing from the follicle wave dynamics studies in PCOS/PMOS (46), it is tempting to hypothesize that the multi-follicular ovarian morphology that emerges in the perimenarcheal years (37, 38) may reflect disorganized follicle growth with sporadic ovulation. Indeed, LH surges are possible in pre- and postmenarche (25); administration of exogenous estradiol that mimics adult-DF estradiol concentrations in premenarcheal adolescent females triggered LH surges, albeit LH surge concentrations were characteristically lower than that of adults, potentially limiting the ability to stimulate follicle rupture (25). That said, ovulation prior to menarche is possible, given a report of pregnancy prior to menses (47).

### Postmenarche

Although menarche can be considered as reproductive maturity, menarche is an endometrial event and does not signify ovulatory competence. Indeed, menstrual irregularity is considered normal within the early postmenarcheal years (33), and ovulatory competence progressively increases through at least five years postmenarche (48, 49). Further, the presence of “regular” menstrual cycles has been associated with minimal, uncoordinated, and coordinated HPO axis activity (22), underscoring that menstruation during the postmenarcheal years is not a reliable proxy for normal HPO axis function. While existing premenarcheal data are generated largely by histological reporting and based on cross-sectional assessments of ovarian morphology, serial ultrasound imaging has been conducted during the postmenarcheal years in three studies (21, 23, 31) which provide insight into DF growth patterns and luteal function in adolescent ovulatory cycles (21, 23, 31), the frequency of anovulatory cycles (21, 23, 31), and their respective endocrine dynamics. To our knowledge, anovulatory follicle dynamics remain to be characterized, therefore we will focus on ovulatory cycle features of the early postmenarcheal years.

Studies investigating growth profiles of the ovulatory follicle in the postmenarcheal adolescent span a wide gynecological age range, which limits inferences into whether certain characteristics may be more pertinent in the less mature versus more mature HPO axis. Cabral et al. monitored DF growth in adolescents approximately 4.6 years postmenarche via every-other-day endovaginal ultrasonography and observed two characteristic growth profiles, defined by a typical (≤16 days) and long (>16 days) follicular phase (FP) (31). DFs emerged at similar times respective to menses and achieved a similar maximal diameter of approximately 18 mm in both typical and long FPs; however, DFs in long FP cycles grew at a slower rate than the DFs of the typical FP among postmenarcheal adolescents (31). The authors also report non-linear growth profiles of the DFs (31), similar to ruminants (50), suggesting conservation of non-linear DF growth profiles in early reproductive maturity. Early to mid FP FSH concentrations were lower in long FPs, leading to the hypothesis that pituitary suppression, rather than ovarian immaturity, may result in slowed follicle growth dynamics (31). Apter et al. conducted daily or every-other-day transabdominal evaluations of follicle growth in conjunction with changes in endocrinology among adolescents 1.4 to 4.7 years postmenarche and reported slower ovulatory follicle growth and ovulation at a smaller diameter later in the FP among adolescents (approximately 17.5 mm, similar to Cabral et al. (31)) versus their adult comparator (approximately 20.2 mm), although the authors did not comment on whether accelerated growth occurred (23), as had been described by Cabral et al. (31). Linear statistical models have also been applied to predict follicle growth dynamics in adolescents 0.4 to 3.5 years postmenarche and predicted both a slower growth rate and subsequent smaller ovulatory diameter (21). Transabdominal scans were conducted on average twice over the menstrual cycle among these participants (21), thus the infrequent sampling may not have enabled a detailed characterization of the DF growth trajectory. Therefore, it remains unclear whether follicle growth dynamics follow a linear or non-linear trajectory as ovulation nears, as is described in the animal models (50), although slower overall growth profiles seem to be in part attributed to lower pituitary stimulation (23–25).

Selection of the ovulatory follicle may also be disrupted in the early postmenarcheal years. Apter et al. describes that although FP rises in FSH occurred in ovulatory adolescents, multiple selectable follicles 8–14 mm were still present on days 12–15 of the menstrual cycle which was characteristically different from their adult counterparts who displayed fewer follicles at this stage in the menstrual cycle that had diameters of 15.4-20.4 mm (23). This contrast demonstrates potential DF incompetence during adolescence to establish LH dependence and promote atresia of the remaining follicles as probable selection nears (23). Therefore, it is plausible that the continued rise and insufficient suppression of FSH during adolescence may explain the polycystic-like ovarian morphology detected in postmenarcheal adolescents (51), presenting the question as to whether the postmenarcheal years may be characterized by uncoordinated, continuous-like follicle dynamics.

Progesterone concentrations are lower during early postmenarche (21, 23), coinciding with the presence of short luteal phases (LPs) in some (21) but not all (23) studies. Luteal insufficiency is common in the postmenarcheal adolescent (21, 23–25), which could lead to aberrant follicle growth profiles and atypically timed ovulations, not unlike what has been observed during the transition to menopause (52). That said, to our knowledge, it is also unclear whether the characteristically lower progesterone described in studies relying strictly on endocrinology (21, 24, 25) are true ovulations versus luteal unruptured follicles (LUFs), which have also been described in postmenarcheal adolescents (21). LUFs can mimic the sonographic appearance of hemorrhagic CLs (53) and thus without serial, intensive ultrasonography, conclusions about structural features could lead to incorrect phenotypic characterization of ovulation.

## Discussion

Serial endocrine and ultrasound techniques applied in animals offer in-depth knowledge about follicle dynamics throughout the lifespan. Serial ultrasonographic and endocrine evaluation of reproductive function is time-consuming and intensive, for both the researchers and the participants. Therefore, it is unsurprising that few data exist. However, such rigorous studies are needed to address fundamental knowledge gaps in human reproductive health. Recent advances in ultrasonographic imaging support the ability for such research methodology. Indeed, the limitations previously encountered in transabdominal imaging presented barriers to reliably resolve antral follicles sufficient for wave dynamic characterization; however, modern imaging technology and techniques offer resolution ability that allows for efficient and accurate visualization of the ovaries in pediatric research (54).

Although ruminant animal models have informed several hypotheses of human follicle wave dynamics (2, 3, 18) and ovarian physiology (55–57), whether the peripubertal ruminant model provides an accurate model for the developmental stage in humans remains to be determined; factors influencing pubertal timing in ruminants (i.e., breed differences, seasonal differences, polyestrus) are not applicable to humans. That said, there are some similarities that should be highlighted. Ruminants exhibit wave-like activity present prior to first ovulation (3, 4, 6, 8, 13), although neuroendocrine and ovarian immaturity manifests as smaller maximal follicle size (3, 4, 6, 8), slower follicle growth rate (8), anovulation (3, 4, 6, 8), and lower relative concentrations of gonadotropins, estradiol, and progesterone (4, 8) that progressively mature following puberty. In prepubertal humans, it remains to be defined whether wave-like patterns are similar to those observed in prepubertal ruminants, however premenarcheal cross-sectional data imply some degree of follicular activity during childhood. Of the available evidence in postmenarcheal adolescents, ovulatory follicle growth patterns and cycle features in the postmenarcheal adolescent are similar to ruminants, with follicles growing slower (21, 23, 31), ovulating at smaller diameters (21, 23, 31), and exhibiting luteal insufficiency (21, 23) and higher rates of anovulatory or aluteal cycles versus the reproductive mature adult (21, 23). Considering the elevated follicle populations described during the peripubertal years (41, 51), AMH may play a supporting role in prolonged FPs through its antagonistic effect on follicle maturation and FSH-sensitivity, resulting in suppressed DF competence and decreased FSH release through increasing the GnRH pulse rate (25, 41). In a departure from ruminant follicle growth dynamics, serial endocrinology and early follicular characterization does not rule out a continuous model of follicular growth during childhood and puberty, with wave-like patterns reflecting a more mature, later stage growth profile.

The need to understand how follicle dynamics evolve during reproductive maturation extends beyond addressing a fundamental knowledge gap. The rise in PCOS/PMOS during adolescence (58) and persistent irregular menstrual cyclicity into adulthood (59) imply that reproductive axis maturation is increasingly aberrant. Hormonal contraceptives, which are commonly used among adolescents (60), have a higher failure rate in adolescents versus adults which is typically attributed to poor compliance (61, 62). However, it’s plausible that contraceptive preparations intended to suppress the mature HPO axis are less effective in the immature HPO axis. Finally, there is a rising incidence of endometrial cancer in US women under 40 years old (63). Whether aberrant reproductive maturation and failure to achieve ovulatory cycles during adolescents results in early and detectable endometrial hyperplasia remains to be elucidated. Overall, a better understanding of ovarian follicle dynamics through puberty and into reproductive maturity will enable earlier detection of reproductive dysfunction, improved contraceptive strategies during adolescence, and more efficacious strategies to mitigate or restore reproductive development when disrupted.
